# Unmasking BCL-2 Addiction in Synovial Sarcoma by Overcoming Low NOXA

**DOI:** 10.3390/cancers13102310

**Published:** 2021-05-12

**Authors:** Carter K. Fairchild, Konstantinos V. Floros, Sheeba Jacob, Colin M. Coon, Madhavi Puchalapalli, Bin Hu, Hisashi Harada, Mikhail G. Dozmorov, Jennifer E. Koblinski, Steven C. Smith, Gregory Domson, Joel D. Leverson, Andrew J. Souers, Naoko Takebe, Hiromichi Ebi, Anthony C. Faber, Sosipatros A. Boikos

**Affiliations:** 1 VCU Philips Institute for Oral Health Research, School of Dentistry and Massey Cancer Center, Perkinson Building Room 4134, 1101 East Leigh St, P.O. Box 980566, Richmond, VA 23298, USA; Fairchildc@vcu.edu (C.K.F.J.); kfloros@vcu.edu (K.V.F.); sjacob2@vcu.edu (S.J.); cmcoon@vcu.edu (C.M.C.); hharada@vcu.edu (H.H.); 2 Department of Pathology, VCU School of Medicine, Richmond, VA 23298, USA; mpuchalapalli@vcu.edu (M.P.); jennifer.koblinski@vcuhealth.org (J.E.K.); steven.c.smith@vcuhealth.org (S.C.S.); 3 Cancer Mouse Models Core, Virginia Commonwealth University, Richmond, VA 23298, USA; bhu@vcu.edu; 4 Department of Biostatistics, Virginia Commonwealth University, Richmond, VA 23298, USA; mdozmorov@vcu.edu; 5 Department of Orthopedic Surgery, Virginia Commonwealth University, Richmond, VA 23298, USA; gregory.domson@vcuhealth.org; 6 AbbVie, 1 North Waukegan Road, North Chicago, IL 60064, USA; joel.leverson@abbvie.com (J.D.L.); Andrew.Souers@abbvie.com (A.J.S.); 7 Division of Cancer Treatment and Diagnosis, National Cancer Institute, NIH, Bethesda, MD 20892, USA; Takeben@mail.nih.gov; 8 Division of Molecular Therapeutics, Aichi Cancer Center Research Institute, Nagoya 464-8681, Japan; hebi@aichi-cc.jp; 9 Precision Medicine Center, Aichi Cancer Center, Nagoya 464-8681, Japan; 10 Division of Advanced Cancer Therapeutics, Nagoya University Graduate School of Medicine, Nagoya 464-8681, Japan; 11 Sarcoma Program, School of Medicine and Massey Cancer Center, Virginia Commonwealth University, Goodwin Research Building, Rm 382, 401 College St. P.O. Box 980037, Richmond, VA 23298, USA

**Keywords:** synovial sarcoma, BCL-2, apoptosis

## Abstract

**Simple Summary:**

Synovial sarcoma is a soft-tissue sarcoma that lacks effective systemic therapy and carries poor prognosis due to frequent late local recurrence and metastases. The cancer is known to be driven in part by increased expression of the pro-survival protein BCL-2. Surprisingly, synovial sarcoma proved resistant to BCL-2 inhibitors in pre-clinical trials. We identified increased activity of a second pro-survival protein, MCL-1, as responsible for this resistance. We showed that co-targeting both BCL-2 and MCL-1 proves to be an effective therapeutic approach both in cell culture and animal models of synovial sarcoma, supporting translation into clinical trials.

**Abstract:**

Synovial sarcoma (SS) is frequently diagnosed in teenagers and young adults and continues to be treated with polychemotherapy with variable success. The SS18-SSX gene fusion is pathognomonic for the disease, and high expression of the anti-apoptotic BCL-2 pathologically supports the diagnosis. As the oncogenic SS18-SSX fusion gene itself is not druggable, BCL-2 inhibitor-based therapies are an appealing therapeutic opportunity. Venetoclax, an FDA-approved BCL-2 inhibitor that is revolutionizing care in some BCL-2-expressing hematological cancers, affords an intriguing therapeutic possibility to treat SS. In addition, there are now dozens of venetoclax-based combination therapies in clinical trials in hematological cancers, attributing to the limited toxicity of venetoclax. However, preclinical studies of venetoclax in SS have demonstrated an unexpected ineffectiveness. In this study, we analyzed the response of SS to venetoclax and the underlying BCL-2 family biology in an effort to understand venetoclax treatment failure and find a therapeutic strategy to sensitize SS to venetoclax. We found remarkably depressed levels of the endogenous MCL-1 inhibitor, NOXA, in SS compared to other sarcomas. Expressing NOXA led to sensitization to venetoclax, as did the addition of the MCL-1 BH3 mimetic, S63845. Importantly, the venetoclax/S63845 combination induced tumor regressions in SS patient-derived xenograft (PDX) models. As a very close analog of S63845 (S64315) is now in clinical trials with venetoclax in AML (NCT03672695), the combination of MCL-1 BH3 mimetics and venetoclax should be considered for SS patients as a new therapy.

## 1. Introduction

Synovial sarcoma (SS) is a rare but deadly soft-tissue sarcoma (STS) with no targeted therapy options. SS accounts for only about 10% of STS, but despite its relative rarity and predominant incidence in pediatric and young adults, 4000 cases are fatal each year [1]. Up to 50–70% of SS patients develop metastasis, which is currently uncurable [2]. Standard of care involves the use of various chemotherapeutic agents but with limited success [3]. SS is a transcription factor-driven cancer, with the SS18-SSX gene fusion pathognomonic for the disease [4]. SS also is known to exhibit high expression of the anti-apoptotic protein BCL-2 [5]; IHC staining for BCL-2 was considered diagnostic for SS prior to the identification of the SS18-SSX gene fusion [5]. The upregulation of BCL-2 makes the BCL-2 inhibitor venetoclax an enticing therapeutic possibility; however, it has been shown to be ineffective as a single agent in SS using preclinical models [6].

The BCL-2 family is a highly conserved family of proteins that functions primarily in and around the mitochondria, involving pro-survival (e.g., BCL-2, BCL-X_L_, and MCL-1), BH3-only pro-death (e.g., BIM, NOXA, PUMA), and terminal-effector molecules (BAK and BAX), the latter of which creates mitochondrial pores upon activation and oligomerization [7]. Under physiologically normal conditions, the interactions amongst the pro-survival and pro-death molecules are maintained at an equilibrium that allows for proper response to intrinsic and extrinsic survival and death signals. In pathologies like cancer, these equilibria typically become unbalanced, shifting towards a pro-survival disposition [7]. The pro-survival proteins bind and sequester the BH3-only activator proteins and prevent the BH3 proteins from activating the effectors BAK and BAX; this serves to neutralize the initiation of mitochondrial pore formation and apoptosis. As such, targeting BCL-2, BCL-X_L_, and MCL-1 via BH3 mimetic drugs, which free BH3-activator proteins to initiate apoptosis, has been a major goal in pharmaceutical development [7]. 

The first BCL-2-selective BH3 mimetic to be tested and approved, venetoclax [8], can be dosed in patients to effectively inhibit BCL-2 and drive robust anti-tumor efficacy [9,10,11]. More recently, selective MCL-1 BH3 mimetics have been developed [12,13,14], and exciting preclinical data has paved the way for clinical testing.

One such molecule is S63845, which has demonstrated preclinical monotherapeutic activity in diverse hematologic cancers as a consequence of the disruption of MCL-1 complexes [12]. Our laboratory [15] and others [13,16] have demonstrated that S63845 can be part of rational combination therapies. Amgen’s MCL-1 inhibitor, AMG 176, was also demonstrated to have preclinical activity across several hematological cancers as well as combination activity with traditional chemotherapeutics [14]. VU661013 is a third MCL-1 inhibitor that, through targeting MCL-1:BIM complexes, effectively combines with venetoclax in mouse models of AML [17]. 

Multiple MCL-1 inhibitors are now in clinical trials, including AMG 176 (NCT02675452), AMG 397 (NCT03465540), AZD-5991, and an inhibitor closely related to S63845 (clinical trial NCT02979366). The relative tolerability of venetoclax makes it an attractive agent to include in combination therapies, and clinical trials exploring concomitant dosing of MCL-1 inhibitors plus venetoclax are underway in several hematologic malignancies [18,19]. 

## 2. Materials and Methods

### 2.1. Cell Lines

SS.PDX and SYO.1 cell lines were cultured in DMEM (Gibco, Gaithersburg, MD, USA) with 10% FBS (Avantor Seradigm, Radnor, PA, USA) and 1 µg/mL penicillin and streptomycin. SW982, ASKA, HS-SY-II, and Yamato cells were grown in RPMI1640 (Lonza Group, Basel, Switzerland) and 1 µg/mL penicillin and streptomycin. Routine mycoplasma testing was performed on all cell lines. The SS.PDX cell line was established ex vivo from the SS patient-derived xenograft model. SYO.1 and SW982 cell lines were kindly provided by Cyril Benes and the Center for Molecular Therapeutics at Massachusetts General Hospital (Boston, MA, USA). ASKA, HS-SY-II, and Yamato cell lines were obtained from Riken Bioresource Center (Kyoto, Japan). 

### 2.2. Dataset Analysis

RNA expression data for sarcoma samples were obtained and analyzed through the R2: Genomic Analysis and Visualization Platform. (http://hgserver1.amc.nl, accessed on September 2019) [20]. RNA expression for genetically modified lines were derived from McBride et al., data accessible at NCBI GEO database, accession GSE108028 [4]. 

### 2.3. Antibodies and Reagents

Primary antibodies used for western blotting were as follows: GAPDH (sc-3233) and MCL-1 (s-19) from Santa Cruz Biotechnology; cleaved PARP1 (5625), cleaved Caspase III (9579), BCL-2 (4223), BCL-X_L_ (2764), BIM (2933), NOXA (14766), and SS18 (70929) from Cell Signaling Technology (Danvers, MA, USA). Secondary antibodies used were anti-mouse IgG (GE Healthcare Life Sciences, Marlborough, MA, USA; NXA931) and anti-rabbit IgG (GE Healthcare Life Sciences; NA934). S63845 was from Abmole (Houston, TX, USA), and venetoclax (ABT-199) was kindly provided by AbbVie Inc (North Chicago, IL, USA). 

### 2.4. Animal Experiments

For the SS patient-derived xenograft model, 5.0 × 10^6^ cells were injected s.c. into each flank of 6–8-week-old NOD/SCID gamma (NSG) mice. The patient-derived xenograft model was acquired from Jackson Laboratories (J000104314). For the SYO.1 xenograft model, 5.0 × 10^6^ cells were injected s.c. into each flank of 6–8-week-old female NOD/SCID gamma (NSG) mice. Treatment began when tumors reached approximately 150 to 200 mm^3^, as calculated using the L*W^2^ *.52 method. Mice were randomized into treatment cohorts, and tumor size was measured at least three times per week for the duration of treatment. Venetoclax was dissolved in 60% Phosal 50 PG, 30% PEG400, and 10% ethanol for a final dosage of 100 mg/kg body weight and administered via oral gavage five days a week [21,22]. Next, S63845 was dissolved in 20% 2-hydroxypropyl-β-cyclodextrin, 25 mM HCL, for a final dosage of 25 mg/kg body weight [12,15]. Then, 25 mg/kg S63845 was administered intravenously every other week on days 1, 2, 3, 6, and 7. All animal experiments were approved by the Virginia Commonwealth University Institution Animal Care and Use Committee (IACUC protocol #AD10001048). 

### 2.5. In Vitro Assays

For CellTiter-Glo (CTG) (Promega, Madison, WI, USA) assays, cells were seeded in quadruplicate in 96-well plates at a concentration of 1.5 × 10^3^ cells/well in 180 µL media. After 24 h, cells were treated with increasing concentrations of ABT-199 and S63845, alone or in combination, for 72 h, at which point 25 µL CTG reagent was added to each well, and plates were read using a Synergy H1 Hybrid microplate reader (BioTek Instruments, Winooski, VT, USA). These experiments were each repeated in triplicate over the course of several weeks. For crystal violet assays, 5.0 × 10^4^ cells were seeded in 6-well plates for 24 h and then treated with ABT-199 and S63845, alone and in combination. Media was changed every other day until the untreated well was fully confluent, at which point cells were stained with 0.1% crystal violet (Sigma Aldrich, St. Louis, MO, USA). For FACS measurements of apoptosis, 2.0 × 10^5^ cells were seeded in 6-well plates and after 24 h, were treated with ABT-199 and S63845 as indicated. After 24 h, cells were collected per BD Biosciences Annexin V/PI staining protocol and then assayed on a Millipore Guava flow cytometer. FACS experiments were conducted in biological triplicate. 

### 2.6. Vector Construction and Establishing Stable Cell Lines

NOXA overexpressing SS.PDX and SYO.1 cell lines were generated as previously described [23]. In brief, SS.PDX and SYO.1 cells were infected with lentivirus-encoding NOXA cDNA, or with an empty vector control. Cells were placed under 400 µg/mL geneticin antibiotic selection for seven days prior to conducting experiments. 

### 2.7. Statistics

For RNA expression data, one-way ANOVA followed by two-tailed *t*-test comparisons were performed using R2: Genomics Analysis and Visualization Platform (http://r2.amc.nl, accessed on September 2019). For the in vivo experiments, Unpaired two-tailed *t*-test comparing tumor volumes, and paired two-tailed *t*-test in comparing mouse weights, were performed using GraphPad Prism version 8.4.2 for macOS (GraphPad Software, San Diego, CA, USA, www.graphpad.com, accessed on September 2019). For CTG experiments, the Bliss additivity method was used to calculate synergy scores [24].

## 3. Results

### 3.1. SS Expresses High Levels of BCL-2 and Low Levels of NOXA

**Figure 1:**
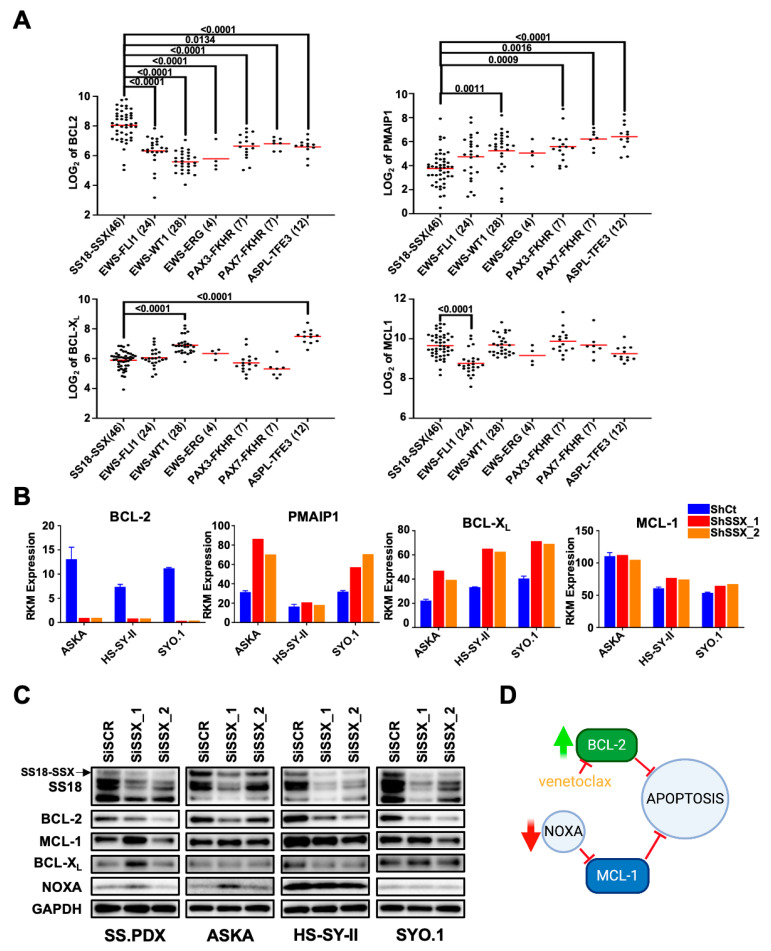
Synovial sarcoma tumors demonstrate increased expression of BCL-2 and decreased expression of NOXA. (**A**) RNA expression levels of key BCL2 family members from fusion-gene driven sarcoma tumor samples. Samples grouped by fusion gene, with sample size shown. Red line indicates mean. Student’s *t*-test was used to compare SS18-SSX to other fusion gene groups; *p* values less than 0.05 are displayed. (**B**) RNA expression levels of key BCL2 family members in ASKA, HS-SY-II, and SYO.1 SS cell lines after shRNA knockdown of SS18-SSX. Two different shRNA constructs are compared to a scramble control. (**C**) Western blot analysis of BCL-2 family proteins in an ex vivo cell line SS.PDX, and cell lines ASKA, HS-SY-II, and SYO.1 after SiRNA knockdown of SS18-SSX. Two separate siRNA constructs are compared to scramble control. (**D**) Our model of BCL-2 family member interactions in synovial sarcoma; the SS18-SSX fusion gene upregulates the anti-apoptotic protein BCL-2. SS cells show low NOXA expression independent of fusion gene expression. Low expression of NOXA, an endogenous inhibitor of the anti-apoptotic protein MCL-1, offers an explanation for SS venetoclax resistance.

Despite high BCL-2 expression being diagnostic for SS, BCL-2 targeting has proven to be an ineffective strategy in preclinical SS models [6]. As the differential expression of anti-apoptotic proteins (e.g., MCL-1 or BCL-X_L_) or pro-apoptotic proteins (e.g., BIM, PUMA, or NOXA) can alter sensitivity to BCL-2 inhibition [8,17,25,26,27], we evaluated the expression of BCL2 family transcripts across multiple sarcoma tumor types [20]. As expected, the SS tumors had a high expression of BCL2 across all samples which, as a group, was significantly higher than any other sarcoma group (Figure 1A). Interestingly, BCL-X_L_ (BCL2L1) expression trended lower in the SS tumors, suggesting it is not a critical factor in venetoclax resistance [21]. In contrast, while MCL1 expression itself trended slightly higher in the SS tumors, strikingly, the endogenous MCL-1 inhibitor NOXA (PMAIP1) was significantly depressed across these samples (Figure 1A). 

The SS18-SSX fusion protein can both activate and repress target gene transcription [28]. The fusion protein interacts with both the BAF enhancer complex and polycomb repressor complex, resulting in genome-wide epigenetic alterations and aberrant expression of many bivalent genes [4,29]. To determine if changes in BCL-2 protein expression levels were directly regulated by the SS18-SSX fusion protein characteristic of SS, we analyzed RNA expression of the BCL-2 family members following knockdown by two short hairpin (SH) constructs against the fusion oncogene [4]. BCL-2 expression was strongly suppressed by both shRNA constructs, suggesting direct and potent regulation of BCL-2 by SS18-SSX (Figure 1B, left panel). Interestingly, knockdown led to either no change or an increase in RNA levels of PMAIP1 (NOXA) (Figure 1B, second panel). BCL2L1-levels were increased while MCL1 mRNA levels were decreased. To determine if protein expression is similarly altered, we knocked down SS18-SSX by two different siRNA constructs [29] targeting the fusion gene in the SS cell lines ASKA, HS-SY-II, and SYO.1. Consistent with the mRNA data, BCL-2 protein expression was markedly decreased after knockdown of SS18-SSX (Figure 1C). However, we saw an increase in NOXA expression only in the ASKA cells, with no change in NOXA expression in the other SS cell lines (Figure 1C). BCL-X_L_ and MCL-1 were largely unchanged across cell lines with either construct. We next analyzed changes in BCL-2 family members after endogenous expression of SS18-SSX in a normal human fibroblast line, CRL-7250 [4]. Consistent with the knockdown experiment, expression of the fusion gene led to a marked increase in BCL2 expression but also led to an increase in PMAIP1, arguing against SS18-SSX having a direct role in downregulation of NOXA (Figure S1a). Next, we compared protein expression levels in five cell lines expressing either the SS18-SSX1 or SS18-SSX2 fusion protein as well as SW982, an atypical SS cell line that does not express the characteristic fusion gene [30,31,32,33] (Table 1, Figure S1b). Compared to the atypical sarcoma, all cell lines expressing the fusion protein showed elevated expression levels of BCL-2. Notably, again with the exception of HS-SY-II, NOXA expression levels were consistently low in all cell lines, regardless of the expression of fusion genes. Taken together, these data indicate that BCL-2 is upregulated by SS18-SSX, while NOXA is likely not regulated by SS18-SSX and is instead low in the progenitor SS cell. Low NOXA expression gives SS progenitors an MCL-1-mediated protection that may prime them for sarcomagenesis, which comes to fruition with the added expression of BCL-2 downstream of SS18-SSX (Figure 1D). 

### 3.2. NOXA Expression Sensitizes SS to Venetoclax

**Figure 2:**
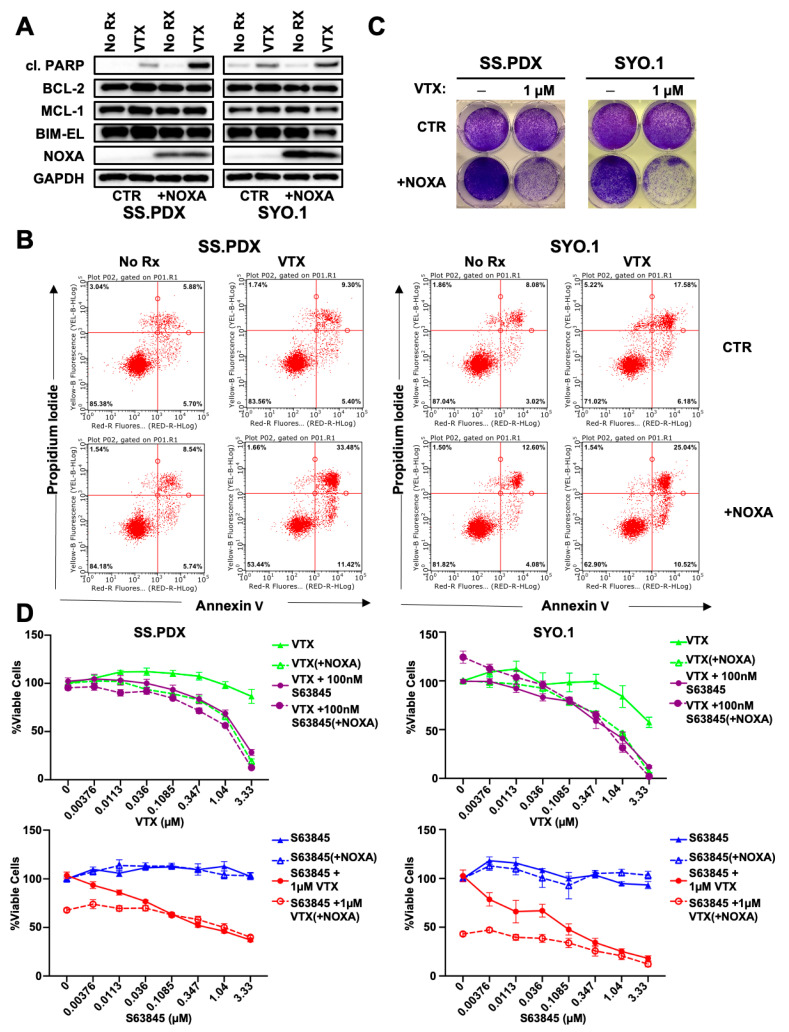
NOXA expression sensitizes SS to venetoclax. (**A**) SS.PDX and SYO.1 cell lines were transfected with a plasmid to overexpress NOXA or an empty vector control (CTR). Following antibiotic-mediated selection, cells were treated overnight with 1 µM venetoclax (VTX). NOXA expression sensitized SS.PDX and SYO.1 cell lines to VTX, as measured by cleavage of PARP1. (**B**) SS.PDX and SYO.1 control and NOXA-overexpressing lines were treated overnight with 1 µM VTX or NT control and analyzed for staining of propidium iodide (y-axis) and annexin-V (x-axis). Graphs were divided into quadrants: bottom-left represents viable cells, top-left necrotic cells, bottom-right early apoptotic cells, and top-right late apoptotic cells. Representative experiment of three biological replicates is displayed. (**C**) Crystal violet staining of SS.PDX and SYO.1 cell lines, with or without expression of NOXA, after 7-day VTX treatment, compared to NT control. (**D**) SS.PDX and SYO.1 control or NOXA-expressing cell lines were treated for 72 h with increasing concentrations of VTX or the MCL-1 inhibitor S63845, alone or in combination. Increasing concentrations of VTX were given alone or combined with constant 100 nM concentration of S63845 (Top panels). Increasing concentrations of S63845 were given alone or in combination with constant 1 µM VTX. Cell viability was analyzed using Cell-Titer Glo (CTG). Error bars indicate standard error of the mean (SEM).

As low NOXA expression has been demonstrated to mediate venetoclax resistance in hematologic cancers [25,34], we hypothesized that low NOXA expression could also explain SS resistance to venetoclax (VTX) (Figure 1D). To test this, we transfected SS.PDX and SYO.1 cell lines with viral particles containing a NOXA-expressing plasmid [23]. Consistent with our hypothesis, NOXA expression sensitized SS cell lines to venetoclax-mediated cell death, as evidenced by increased PARP cleavage and increased surface expression of phosphatidylserine, the characteristic markers of the apoptotic cell death process (Figure 2A,B, Figure S2a). These differences were also appreciated over seven-day treatment followed by crystal violet staining (Figure 2C). As MCL-1 BH3 mimetics are now in clinical trials, we asked if the addition of MCL-1 BH3 mimetic S63845 [12] could phenocopy the effects of inducing NOXA. When treated with clinically relevant concentrations of S63845, neither SS cell lines nor NOXA-expressing SS cell lines showed reductions in cell viability (Figure 2D, bottom panels). However, NOXA-expressing cells, or cells treated with S63845, showed equivalent and marked decreases in cell viability when treated with increasing concentrations of venetoclax (Figure 2D, top panels), despite the latter showing no monotherapy activity (Figure 2D, top panels) [6]. Taken together, these results indicate that low NOXA expression associates with a protective effect of MCL-1 to mediate venetoclax resistance in SS, and this protection can be reversed via pharmaceutical MCL-1 inhibition.

### 3.3. SS Is Sensitive to Combined Inhibition of BCL-2 and MCL-1 In Vitro

**Figure 3:**
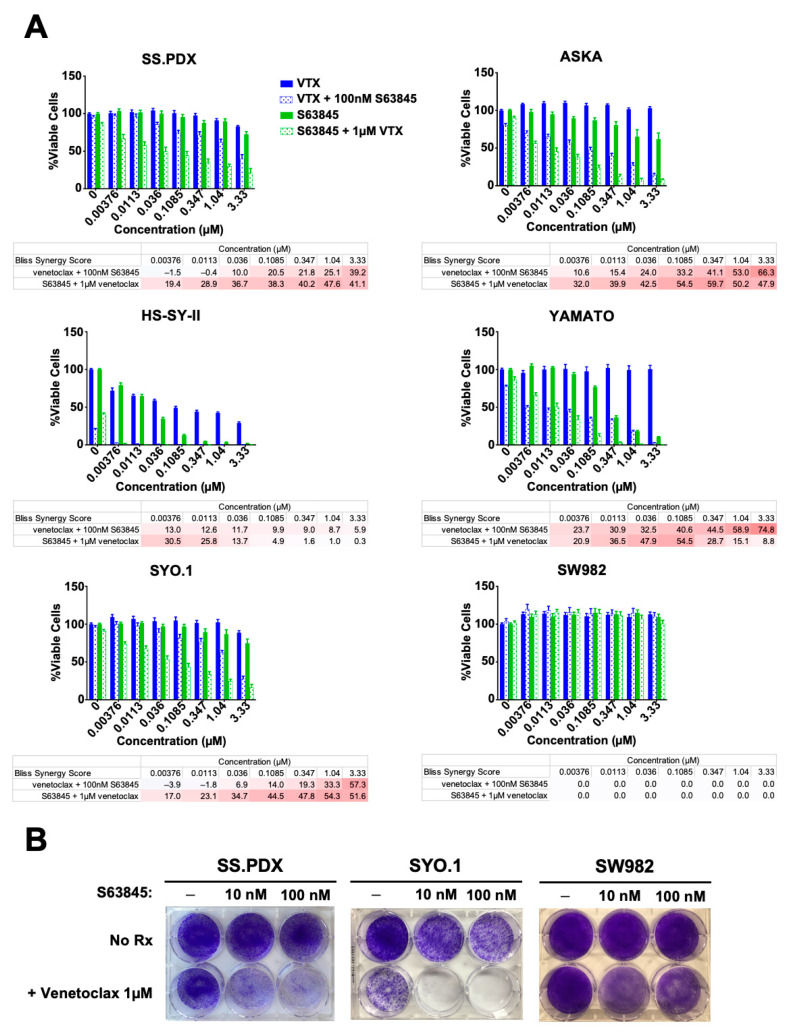
The MCL-1 inhibitor S63845 and VTX combine synergistically to reduce cell viability. (**A**) Scheme 1. and the atypical SS cell line SW982 were treated with increasing concentrations of VTX (blue bars) or S63845 (green bars), alone (solid bars) or in combination (hollow bars). Cells were treated for 72 h and cell viability was measured using CTG. Error bars indicate SEM. Bliss synergy scores are displayed, ranging from –100 (blue) to +100 (red). (**B**) Crystal violet staining of SS.PDX, SYO.1, and SW982 cells after 7-day treatment with S63845 or VTX, alone or in combination, at the concentrations indicated.

After observing that NOXA expression sensitized SS cell lines to venetoclax and S63845 similarly sensitized SS cell lines, we chose to expand our investigation into the efficacy of venetoclax in combination with S63845 as a therapy for SS. We exposed a panel of SS cell lines to venetoclax and S63845, both alone and in combination. After 72 h of treatment, cell viability was assessed using CTG assay. Each drug showed limited efficacy as a single agent, yet strong synergistic cell killing in combination across all five cell lines expressing the SS18-SSX fusion gene (Figure 3A). In contrast, the atypical synovial sarcoma SW982, which expresses neither the SS18-SSX oncogene nor high levels of BCL-2, was resistant to the combination (Figure 3A). Importantly, and consistent with our hypothesis, only HS-SY-II, the sole SS line tested that did not have depressed levels of NOXA, showed moderate single-agent sensitivity to venetoclax (Figure 3A, Figure S1b). 

We also conducted seven-day treatments to assess SS response to the combined treatment (Figure 3B). Because we noticed strong synergistic activity consistently across cell lines with S63845 concentrations at or below 100 nM, a relatively low dose of S63845 [15], we chose to continue our investigation at this dose. As in the first CTG assays, SS cell lines expressing the fusion-oncogene showed response to the combined venetoclax/S63845 drug treatment while showing little response to single-agent venetoclax. Again, the atypical SS cell line SW982 showed no response to the combination therapy. These data demonstrate that, in SS driven by the SS18-SSX fusion gene, concomitant MCL-1 and BCL-2 inhibition is effective in reducing cell viability in vitro.

### 3.4. The Combination of Venetoclax and S63845 Liberates BIM and Induces Apoptosis

**Figure 4:**
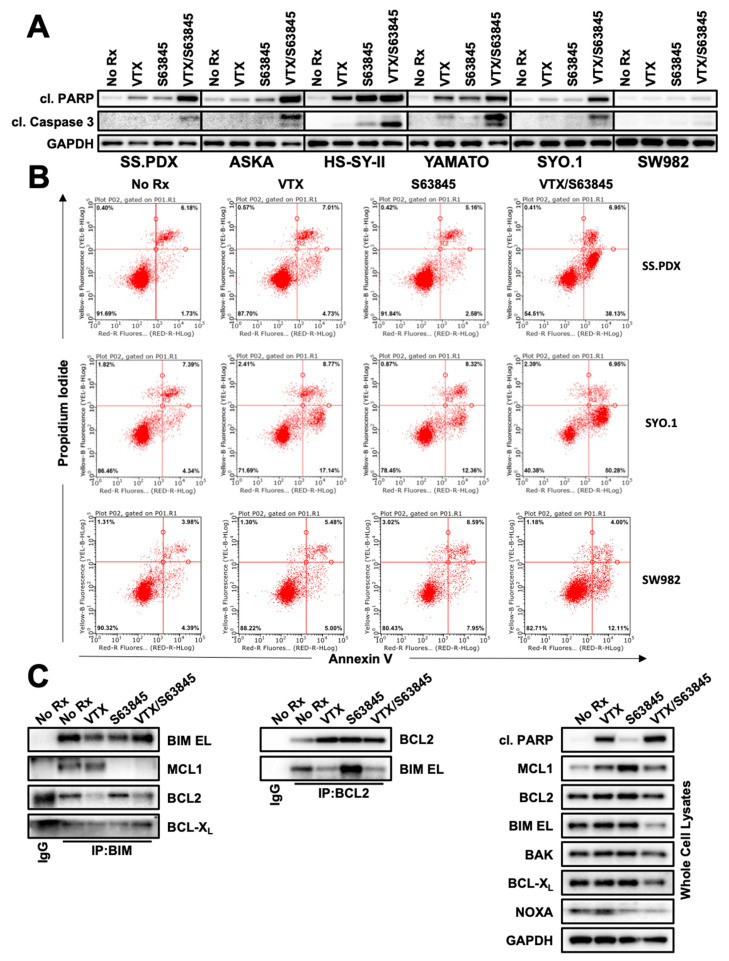
S63845 and VTX combine to induce apoptosis in SS cell lines. (**A**) Western blot analysis of key apoptotic markers cleaved PARP1 and cleaved caspase 3 after overnight treatment with 1 µM VTX, 100 nM S63845, or combination, in SS.PDX, ASKA, HS-SY-II, Yamato, and SW982 cell lines. (**B**) SS.PDX, SYO.1, and SW982 cell lines were treated overnight with 1 µM VTX, 100 nM S63845, or the combination and analyzed for staining of propidium iodide (y-axis) and annexin-V (x-axis). Graphs were divided into quadrants: bottom-left represents viable cells; top-left necrotic cells; bottom-right early apoptotic cells; and top-right late apoptotic cells. Representative experiment of three biological replicates is displayed. (**C**) Co-immunoprecipitation shows VTX and S63845 disrupt pro-apoptotic protein binding to BCL-2 and MCL-1, respectively; left panel: pulldown with pro-apoptotic protein BIM; middle panel: pulldown with BCL-2; right panel: whole cell lysates.

BCL-2 and MCL-1 are critical anti-apoptotic proteins and BH3 mimetics against these two molecules that induce apoptosis [34,35]. We found that 24-h treatment of cells with venetoclax and S63845 resulted in the cleavage of PARP1 (Figure 4A) with only modest amounts of cleaved PARP1 in the single agent-treated cells. Additionally, cleaved caspase 3 (CC3) was expressed in a similar pattern after these drug treatments. Consistent with the viability assays, the atypical SS SW982 showed little cleavage of either PARP1 or caspase 3 (Figure 4A). We next evaluated the externalization of phosphatidylserine (Figure 4B, Figure S2b), a characteristic marker of apoptosis. Again, we found marked apoptosis only in the combination-treated SS cell lines; the atypical SS cell line SW982 showed no increase in apoptosis with combination treatment. These data, together with PARP1 and caspase 3 cleavage, confirmed robust induction of apoptosis by the combination of venetoclax and S63845, specifically in SSX-SS18-driven SS.

These data prompted further investigation into how venetoclax and S63845 interacted to increase the amount of apoptosis. BIM is a pro-apoptotic protein that mediates venetoclax-mediated cell killing [7,21]. Analysis of BIM complexes showed a modest but detectable increase in MCL1:BIM complexes after venetoclax treatment, whereas BCL-2:BIM complexes were increased by S63845 (Figure 4C). Upon combination treatment, BIM was released from both BCL-2 and MCL-1 (Figure 4C). 

### 3.5. SS Mouse Models Are Sensitive to Venetoclax-S63845 Combination In Vivo

**Figure 5:**
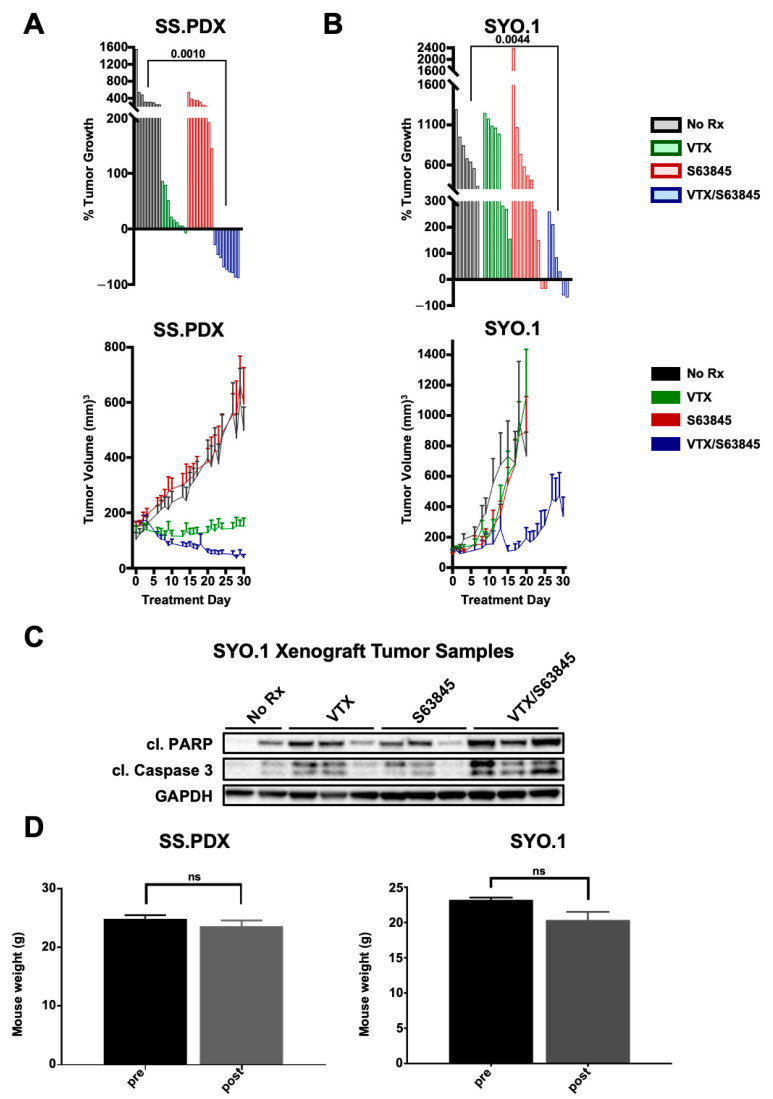
Venetoclax and S63845 combine to inhibit tumor growth in vivo. Mice were injected with 5 × 10^6^ cells from a patient-derived xenograft model of SS (**A**) or from the SYO.1 cell line model (**B**) to generate tumors. Tumor size was measured by caliper and tumor volume calculated by L*W^2^ *.52. As tumors approached a volume of approximately 150 mm^3^, mice were randomized to no treatment, VTX, S63845, or a combination of both drugs. Mice in the VTX and combination groups received daily 100 mg/kg VTX orally for four weeks. S63845 and combination groups received 25 mg/kg S63845 IV daily on weeks 1 and 3, with a treatment holiday on weeks 2 and 4. ((**A**,**B**) (top)): Waterfall plot showing change in tumor volume of no treatment (black), VTX (green), S63845 (red), and combination (blue) groups. Each bar represents an individual tumor. For SS.PDX, initial tumor sizes were compared to final tumor volume after 30-day treatment. Because SYO.1 tumors tended to grow much faster, initial volume was compared to tumor volume after 20 days. (**A**,**B**) *p* values, indicated on graph, were calculated using Student’s *t*-test. (bottom): Average change in tumor growth over time. Error bars indicate SEM. (**C**) Western blot analysis of apoptotic markers cleaved PARP1 and cleaved caspase 3. Representative tumor lysates from no treatment, VTX, S63845, and combination groups are displayed. (**D**) Neither SS.PDX nor SYO.1 combination groups showed a significant decrease in weight over the treatment regimen.

We next assessed the anti-tumor effect of venetoclax and S63845 in a patient-derived xenograft (PDX) model of SS. Mice were treated with 100 mg/kg of venetoclax [22] delivered once daily, 25 mg/kg of S63845 [15,16] delivered once daily on alternating weeks, or a combination of both therapies. While venetoclax alone showed tumor growth inhibition, only the combination therapy induced tumor regressions (Figure 5A), consistent with the efficacy of the combination in vitro. In fact, all nine tumors decreased in size following the combination therapy (Figure 5A, top). We expanded our in vivo study to an SYO.1 cell-line xenograft model. In general, the cell-line xenograft tumors grew much faster compared to the PDX tumors, and thus comparisons were made at 20d post-initiation of treatment, at which point the majority of mice in the control and single-agent groups were euthanized due to tumor burden. The combination significantly reduced tumor growth compared to either single-agent therapy or control groups (Figure 5B). Analysis of SYO.1 tumor lysates showed increased cleavage of PARP1 and caspase 3 in the combination group compared to the vehicle or single-agent groups (Figure 5C). We saw no significant decrease in mouse weight over the course of treatment in either experiment, indicating tolerability of the combination treatment (Figure 5D). Overall, these data support the in vitro studies, demonstrating the efficacy and tolerability of the venetoclax-S63845 combination in SS models.

## 4. Discussion

SS accounts for ~10% of pediatric STS and, due to its metastatic potential and the very limited efficacy of chemotherapies, is often fatal [36]. The known reliance of SS on SS18-SSX to drive and maintain tumorigenicity renders this fusion gene an appealing therapeutic target; however, no direct small-molecule inhibitors have yet been developed [4,29]. Moreover, the use of drugs targeting epigenetic pathways has offered disappointing results. For instance, EZH2 inhibitors such as tazemetostat have demonstrated activity in SS cell lines, leading to enrollment of pediatric and adult SS patients in phase I and II trials with this agent [37,38]. Unfortunately, no objective benefit was demonstrated [39]. Similarly, several surface markers have been associated with SS, notably NY-ESO-1. Preclinical and early clinical trials investigating immunotherapies offer some promise; however, clinical data has yet to mature and immuno-oncology therapy does not offer durable responses in all patients [40,41]. We chose to focus on other characteristic protein expression signatures in the hope of identifying an exploitable therapeutic target. Because SS is known to overexpress BCL-2 and venetoclax-based combination therapies are very effective across multiple hematological cancers, we chose to focus on deciphering the role of BCL-2 family members in this cancer. Previous work has shown SS cell lines to be resistant to single-agent treatment with venetoclax [6]. Here, we show inhibition of MCL-1 is sufficient to sensitize SS cell lines to venetoclax in vitro, and, critically, the MCL-1 inhibitor S63845 and venetoclax synergize to induce tumor regression in vivo. Our analysis included cell lines derived from patients of both sexes, with different SS18-SSX fusions and histologic subtypes, and included samples from both primary and metastatic sites (Table 1). 

Venetoclax is effective in the clinic as a monotherapy against chronic lymphocytic leukemia (CLL), which expresses BCL-2 at high levels [6], as well as in AML [17] and other hematologic malignancies [42]. In solid tumors, we have found that venetoclax has preclinical activity in SCLC where high BCL-2 expression serves as a biomarker [22]; based on these data, an early-phase clinical trial will soon be enrolling to evaluate the combination of venetoclax and irinotecan. However, we have found that a subset of SCLC lines with high BCL-2 expression are resistant to venetoclax [22]. In a similar vein, Jones and colleagues demonstrated venetoclax was ineffective across preclinical models of SS despite high BCL-2 expression and a vital role for BCL-2 in sarcomagenesis [6]. Similarly, we found venetoclax to be ineffective against SS models. 

MCL-1 is among the most well-characterized resistance factors for venetoclax. As a structurally related anti-apoptotic BCL-2 family member, MCL-1 can bind to pro-apoptotic factors released from BCL-2, such as BIM and BAK, thus preventing venetoclax-mediated apoptosis [21,43,44]. NOXA is a BH3-only protein that can selectively bind and neutralize MCL-1, effectively precluding it from sequestering other pro-apoptotic proteins. Low NOXA expression is sufficient to cause resistance to BCL-2 inhibition in SCLC [23] and neuroblastoma [21]. In these settings, the lack of NOXA leaves a greater proportion of MCL-1 free to bind to and sequester pro-death proteins [23]. This then sensitizes cells to treatment with a MCL-1 inhibitor, which can liberate the pro-apoptotic factors leading to induction of apoptosis. [15] Here, we demonstrate that SS has depressed levels of NOXA and that treatment with an MCL-1 inhibitor in combination with venetoclax affords highly synergistic killing of SS cells in vitro and tumor regression in vivo.

SS has long been known to characteristically overexpress BCL-2 [6]; SS18-SSX has been shown to upregulate BCL-2, though the precise mechanism remains elusive [45]. While we confirm that SS18-SSX expression positively regulates BCL-2 expression, we did not find evidence that NOXA was regulated by SS18. Induction of SS in murine tissues was only possible when SS18-SSX was induced in a specific myoblastic lineage, demonstrating that the fusion gene requires a specific cellular environment to drive malignant transformation [46]. We hypothesize that SS emerges from a cell with low NOXA expression, which affords MCL-1-mediated protection early in tumorigenesis. Upon expression of SSX-SS18, BCL-2 expression increases, conferring MCL-1- and BCL-2-mediated protection from apoptosis. 

Multiple MCL-1 inhibitors are now being evaluated in early clinical trials [47]. In addition, phase 1 trials investigating venetoclax in combination with S64315 (NCT03672695), AMG 176, or AZD5991 are underway in refractory hematologic malignancies. Recent reports have provided optimism that co-targeting BCL-2 and MCL-1 may be tolerated generally [14,48]. However, conditional deletion of MCL-1 in mice induced heart failure in a BAK- and BAX-dependent process, raising concerns for cardiac toxicity with MCL-1 inhibitors [49]. Interestingly, we have shown that low doses of S63845 disrupt MCL-1:BIM but not MCL-1:BAK complexes [15] and herein demonstrate robust combination efficacy with venetoclax. It is worth investigating further how disruption of MCL-1:BIM and MCL-1:BAK complexes contribute to MCL-1 inhibitor toxicity generally and, in particular, in cardiomyocytes.

## 5. Conclusions

Despite high levels of BCL-2 in SS, the activity of venetoclax in preclinical models of SS has been disappointing. This study demonstrates that suppressed NOXA expression leads to an MCL-1-mediated resistance to venetoclax in SS, thus revealing an Achilles’ heel that can be exploited by concomitant treatment with an MCL-1 inhibitor. Simultaneous disruption of both BCL-2 and MCL-1 via small-molecule inhibitors induces robust regression of tumors in a SS PDX model and cell-line xenograft model as well as multiple cell lines derived from both primary and metastatic disease. Given the lack of therapeutic options for SS, these data provide the rationale for clinical evaluation of this combination.

## Figures and Tables

**Table 1:** Summary of cell lines.

Cell Line	SS.PDX	ASKA	HS-SY-II	Yamato	SYO.1	SW982
Age	21	27	Adult	30	19	25
Sex	Male	Male	Male	Male	Female	Female
Mutation	SS18-SSX1	SS18-SSX1	SS18-SSX1	SS18-SSX1	SS18-SSX2	BRAF V600E
Histology	Monophasic	Biphasic	Monophasic	Biphasic	Biphasic	Biphasic
Cell line Source	Metastatic site	Metastatic site	Metastatic site	Primary site	Primary site	Primary site

## Data Availability

RNA expression data for sarcoma samples were obtained and analyzed through the R2: Genomic Analysis and Visualization Platform. (http://hgserver1.amc.nl, accessed on September 2019) [20]. RNA expression for genetically modified lines in Figure 1B and Supplementary Figure S1A were derived from McBride et al., data accessible at NCBI GEO database, accession GSE108028 [4].

## Supplementary Materials

The following are available online at https://www.mdpi.com/article/10.3390/cancers13102310/s1, Figure S1: (**a**) RNA expression levels of key BCL-2 family members are compared in a naiïve CRL-7250 fibroblast line and after expression of SS18 or SS18-SSX. (**b**) Western blot expression levels of BCL-2 family members in the ex vivo SS line SS.PDX; SS cell lines ASKA, HS-SY-II, Yamato, and SYO.1; and the atypical SS cell line SW982, which lacks the fusion gene. Figure S2: (**a**) Average of annexin V-positive cells transduced with empty vector control (CTR) or transduced with a NOXA-expressing vector after treatment with venetoclax. Average of three biological replicates corresponding to representative data displayed in Figure 2B. (**b**) Average of annexin V-positive cells after treatment with venetoclax or S63845 alone and in combination. Average of three biological replicates corresponding to representative data displayed in Figure 4B.
