# Supplementary material for: Unmasking BCL-2 Addiction in Synovial Sarcoma by Overcoming Low NOXA
Source: Cancers (Basel). 2021 May 12;13(10):2310. doi: 10.3390/cancers13102310 (PMC8150384; doi:10.3390/cancers13102310)

Figure 1C

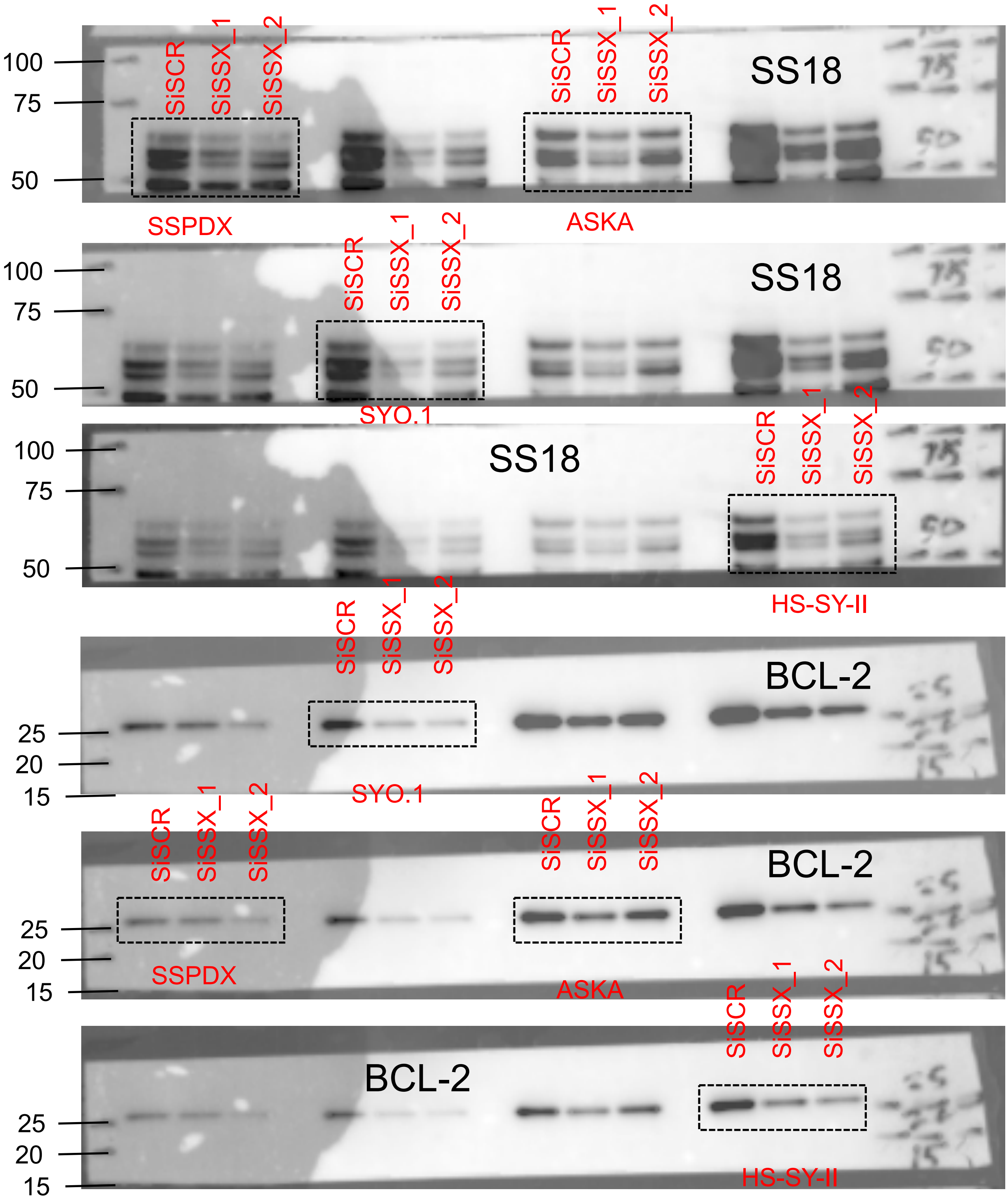

SS18
SiSCR
SiSSX_1
SiSSX_2
100
75
50
SSPDX
ASKA
SYO.1
HS-SY-II
BCL-2
25
20
15

Figure 1C

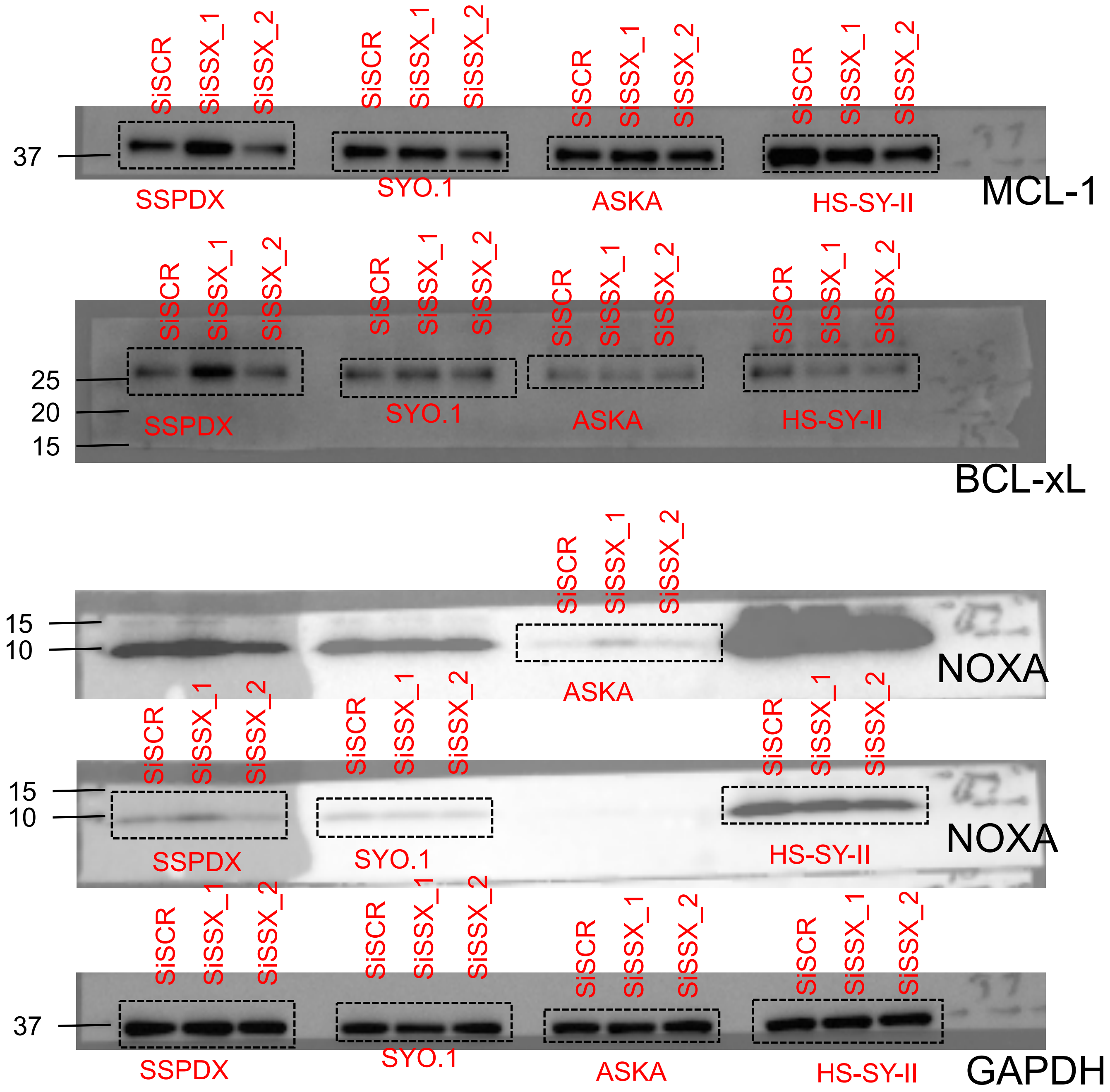
SiSCR
SiSSX_1
SiSSX_2
37
SSPDX
SYO.1
ASKA
HS-SY-II
MCL-1
25
20
15
BCL-xL
15
10
NOXA
NOXA
GAPDH

Figure 2A

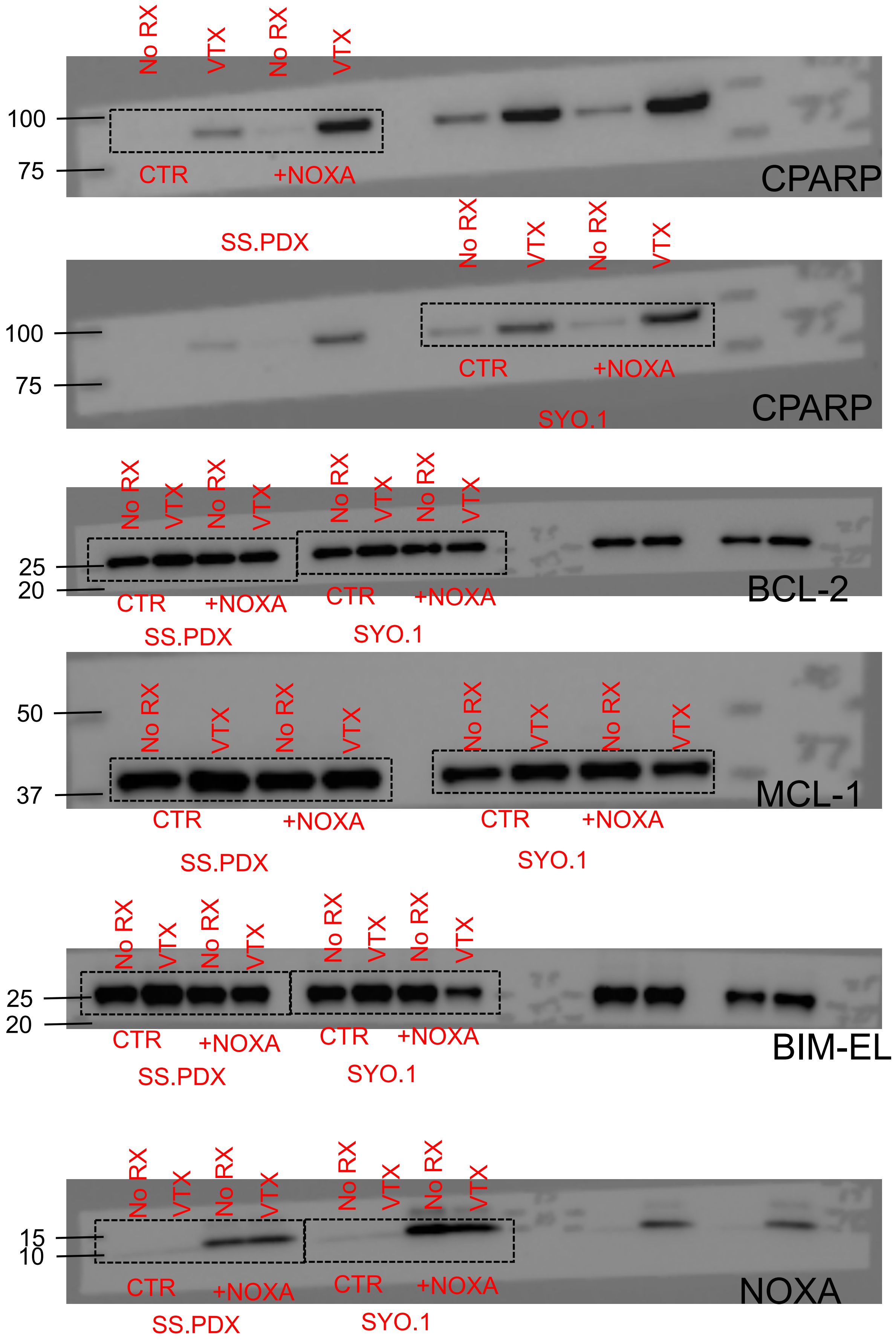

No RX
VTX
No RX
VTX
100
75
CTR
+NOXA
CPARP
SS.PDX
No RX
VTX
No RX
VTX
100
75
CTR
+NOXA
CPARP
SYO.1
No RX
VTX
No RX
VTX
No RX
VTX
No RX
VTX
25
20
CTR
+NOXA
SS.PDX
CTR
+NOXA
SYO.1
BCL-2
No RX
VTX
No RX
VTX
No RX
VTX
No RX
VTX
50
37
CTR
+NOXA
SS.PDX
CTR
+NOXA
SYO.1
MCL-1
No RX
VTX
No RX
VTX
No RX
VTX
No RX
VTX
25
20
CTR
+NOXA
SS.PDX
CTR
+NOXA
SYO.1
BIM-EL
No RX
VTX
No RX
VTX
No RX
VTX
No RX
VTX
15
10
CTR
+NOXA
SS.PDX
CTR
+NOXA
SYO.1
NOXA

Figure 2A

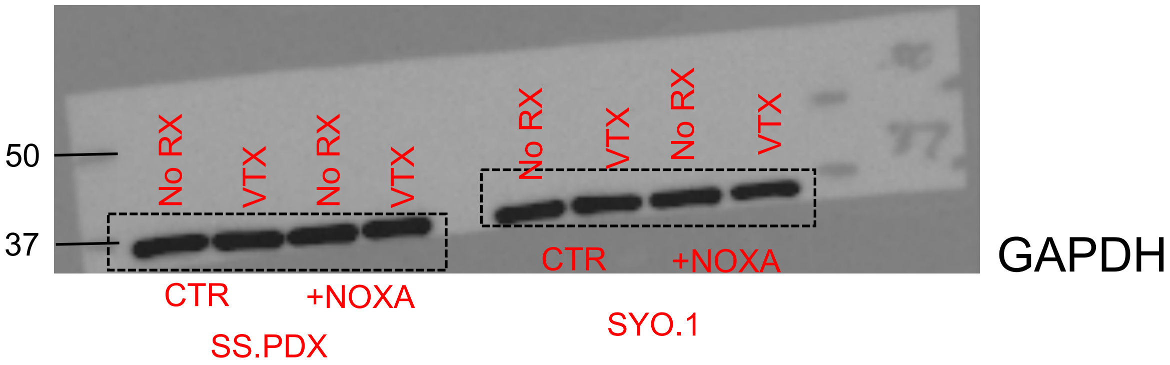

50
37
No RX
VTX
No RX
VTX
No RX
VTX
No RX
VTX
CTR
+NOXA
CTR
+NOXA
GAPDH
SS.PDX
SYO.1

Figure 4A

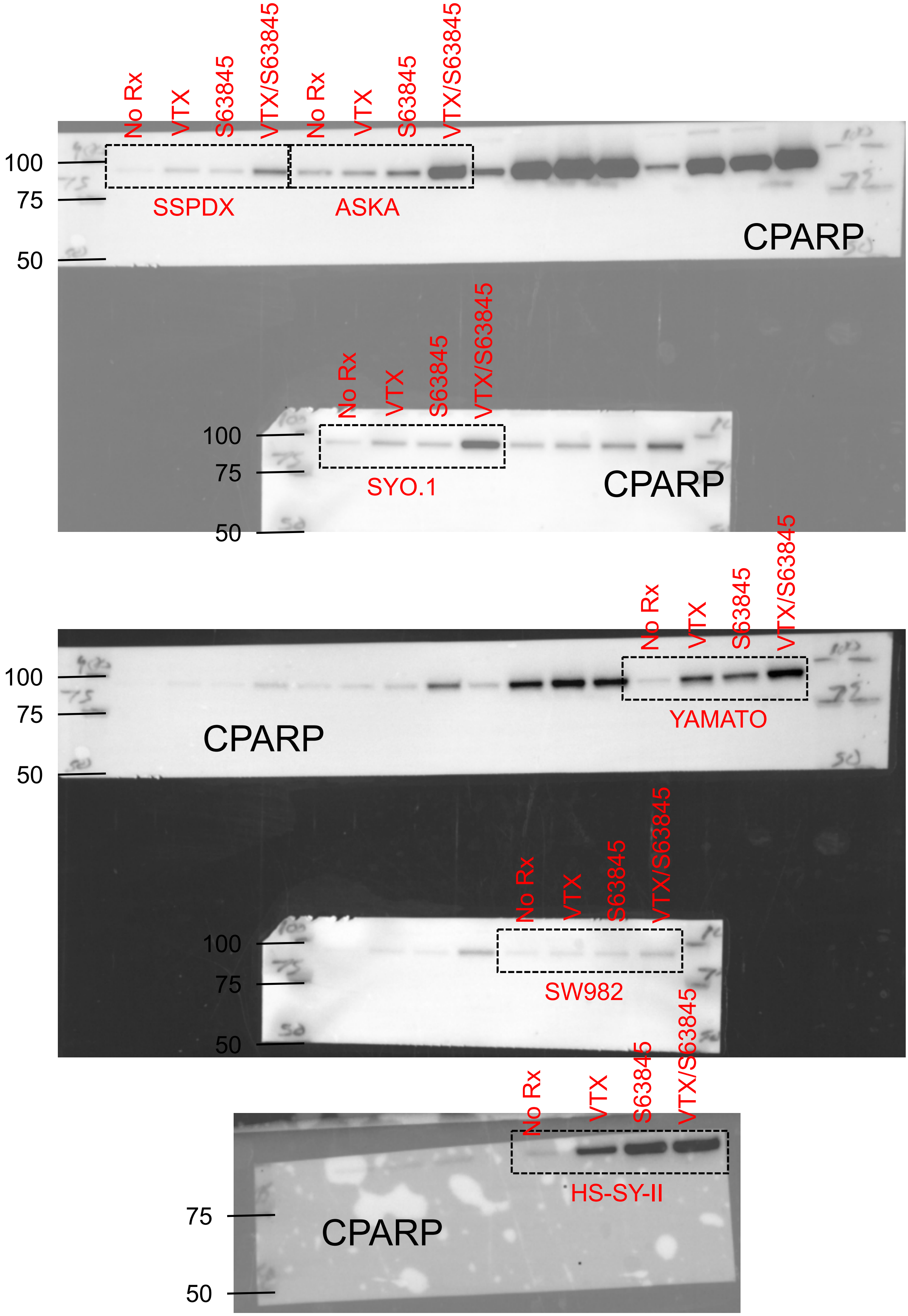
No Rx
VTX
S63845
VTX/S63845
No Rx
VTX
S63845
VTX/S63845
100
75
50
SSPDX
ASKA
CPARP
No Rx
VTX
S63845
VTX/S63845
100
75
50
SYO.1
CPARP
No Rx
VTX
S63845
VTX/S63845
100
75
50
YAMATO
CPARP
No Rx
VTX
S63845
VTX/S63845
100
75
50
SW982
No Rx
VTX
S63845
VTX/S63845
75
50
HS-SY-II
CPARP

Figure 4A

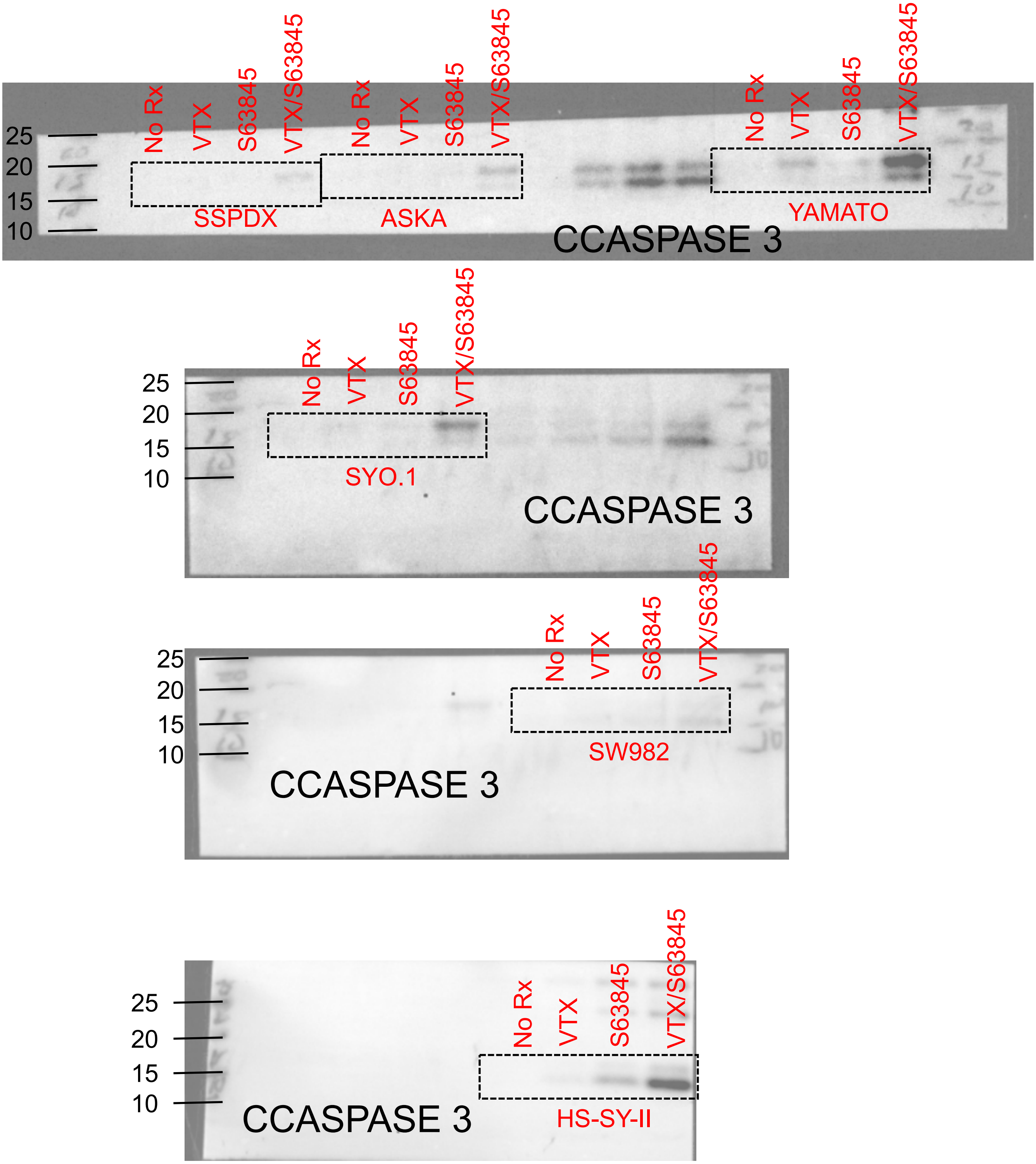

25
20
15
10
No Rx
VTX
S63845
VTX/S63845
No Rx
VTX
S63845
VTX/S63845
No Rx
VTX
S63845
VTX/S63845
SSPDX
ASKA
YAMATO
CCASPASE 3
25
20
15
10
No Rx
VTX
S63845
VTX/S63845
SYO.1
CCASPASE 3
25
20
15
10
No Rx
VTX
S63845
VTX/S63845
SW982
CCASPASE 3
25
20
15
10
No Rx
VTX
S63845
VTX/S63845
CCASPASE 3
HS-SY-II

Figure 4A

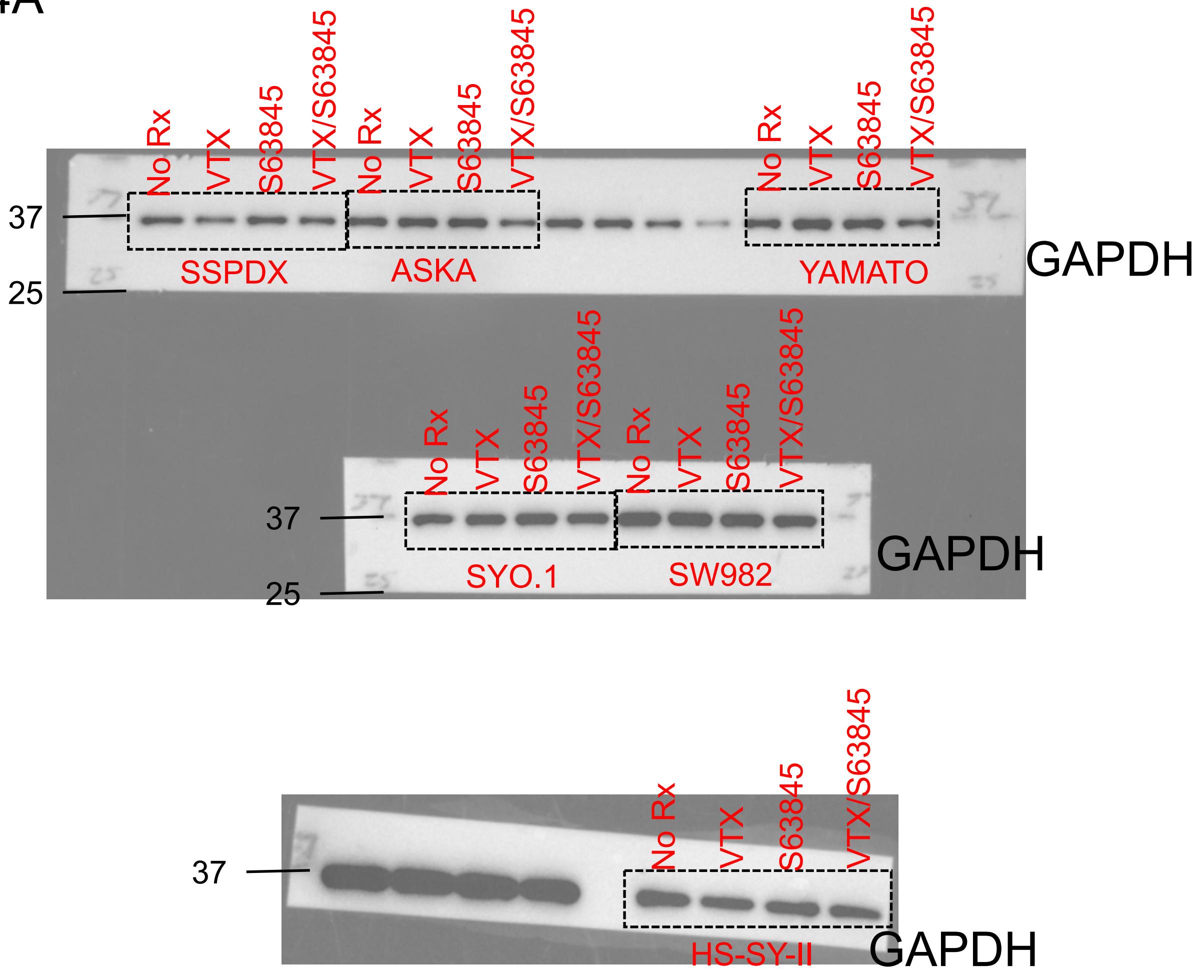

No Rx
VTX
S63845
VTX/S63845
No Rx
VTX
S63845
VTX/S63845
No Rx
VTX
S63845
VTX/S63845
37
25
SSPDX
ASKA
YAMATO
GAPDH
No Rx
VTX
S63845
VTX/S63845
No Rx
VTX
S63845
VTX/S63845
37
25
SYO.1
SW982
GAPDH
No Rx
VTX
S63845
VTX/S63845
37
HS-SY-II
GAPDH

Figure 4C

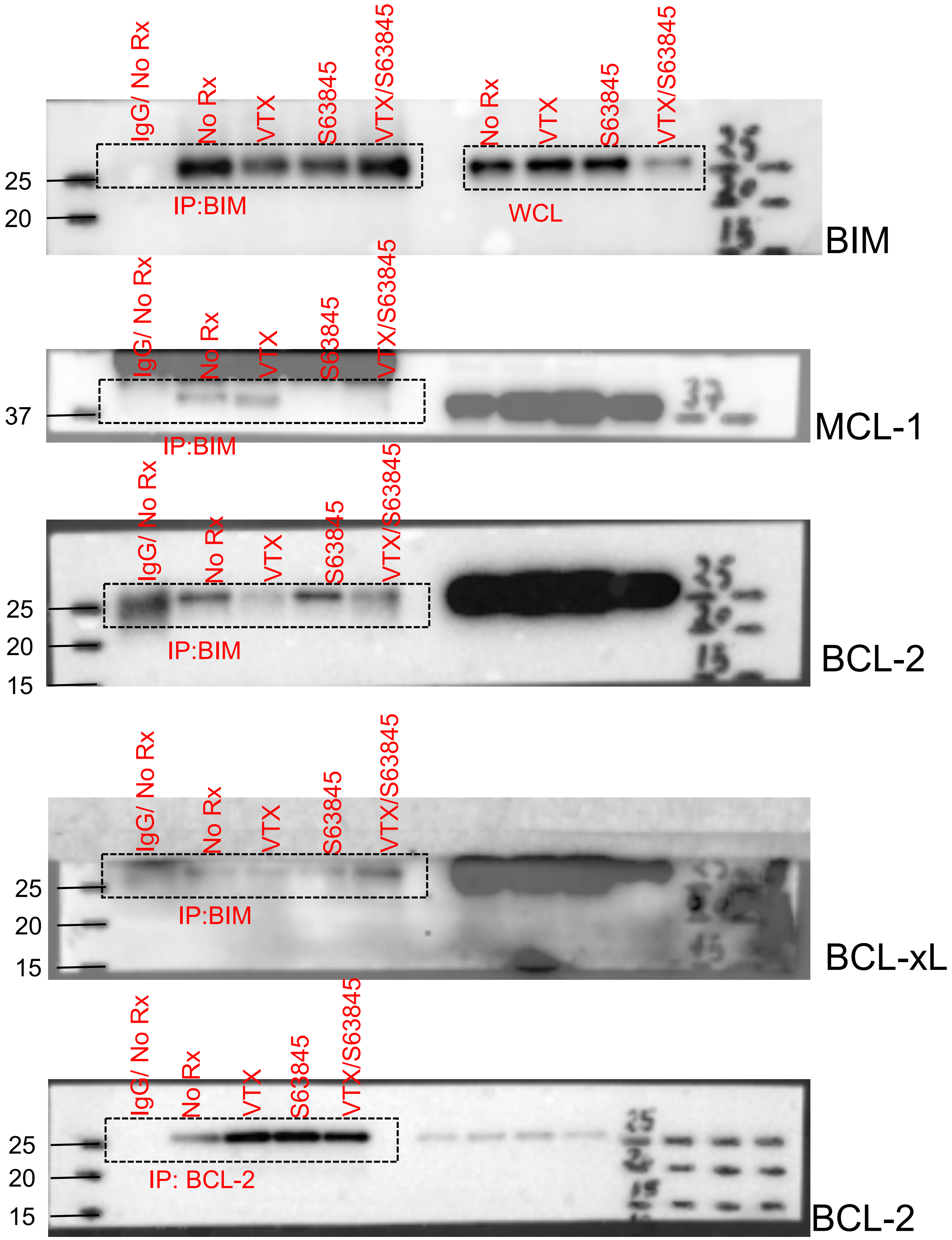

IgG/ No Rx
No Rx
VTX
S63845
VTX/S63845
No Rx
VTX
S63845
VTX/S63845
25
20
IP:BIM
WCL
BIM
IgG/ No Rx
No Rx
VTX
S63845
VTX/S63845
37
IP:BIM
MCL-1
IgG/ No Rx
No Rx
VTX
S63845
VTX/S63845
25
20
15
IP:BIM
BCL-2
IgG/ No Rx
No Rx
VTX
S63845
VTX/S63845
25
20
15
IP:BIM
BCL-xL
IgG/ No Rx
No Rx
VTX
S63845
VTX/S63845
25
20
15
IP: BCL-2
BCL-2

Figure 4C

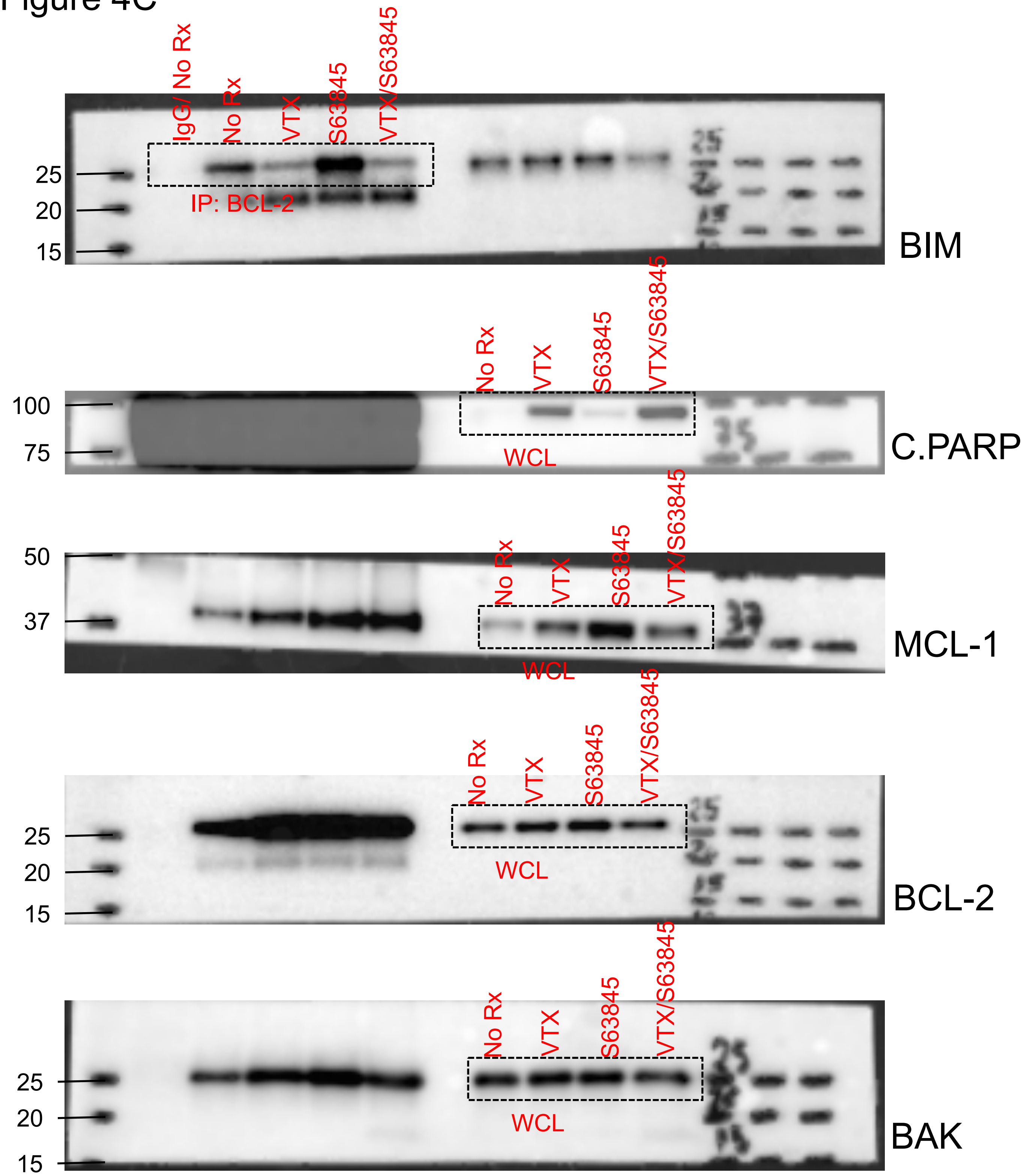
IgG/ No Rx
No Rx
VTX
S63845
VTX/S63845
25
20
15
IP: BCL-2
BIM
No Rx
VTX
S63845
VTX/S63845
100
75
WCL
C.PARP
No Rx
VTX
S63845
VTX/S63845
50
37
WCL
MCL-1
No Rx
VTX
S63845
VTX/S63845
25
20
15
WCL
BCL-2
No Rx
VTX
S63845
VTX/S63845
25
20
15
WCL
BAK

Figure 4C

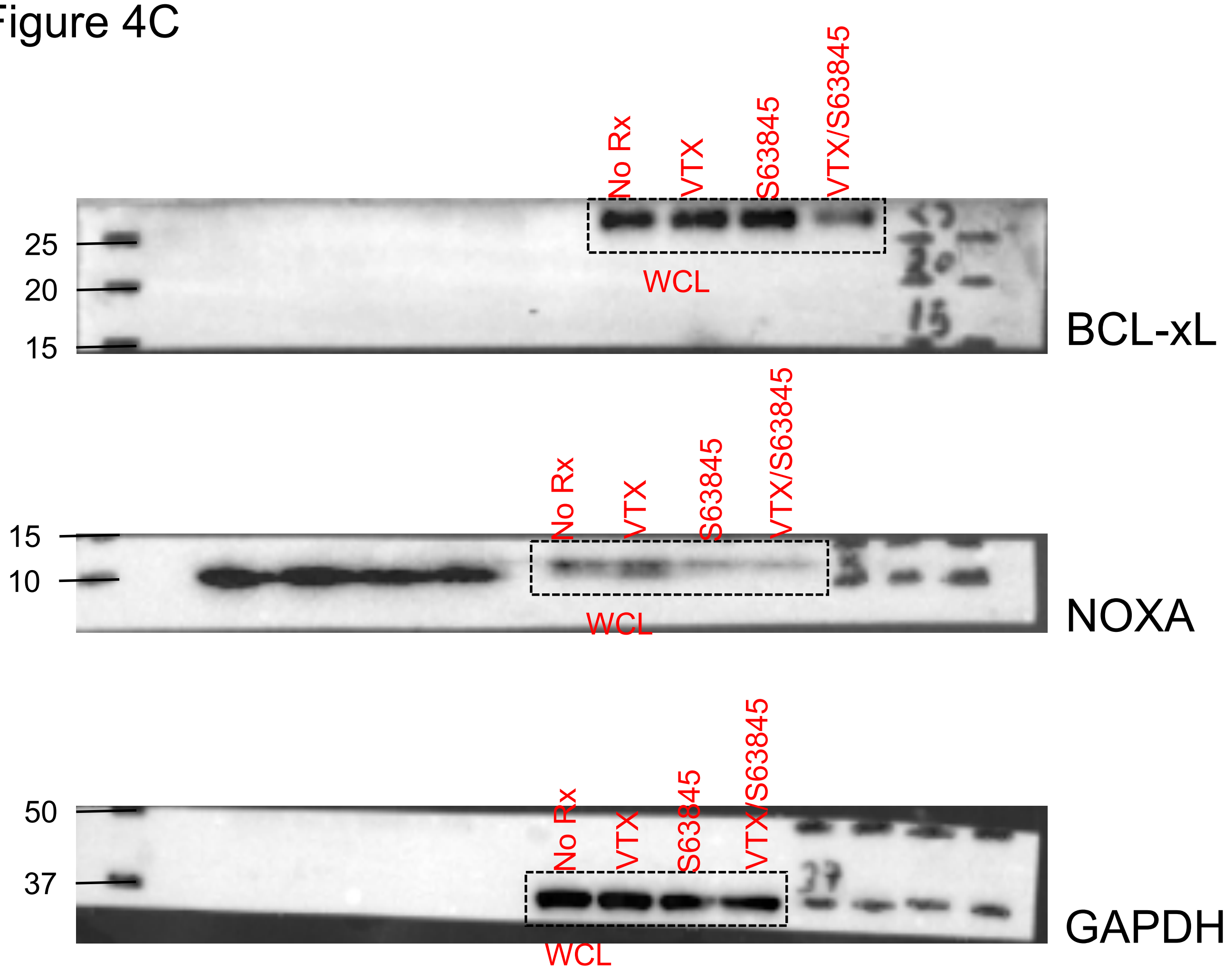
No Rx
VTX
S63845
VTX/S63845
25
20
15
WCL
BCL-xL
No Rx
VTX
S63845
VTX/S63845
15
10
WCL
NOXA
No Rx
VTX
S63845
VTX/S63845
50
37
WCL
GAPDH

Figure 5C

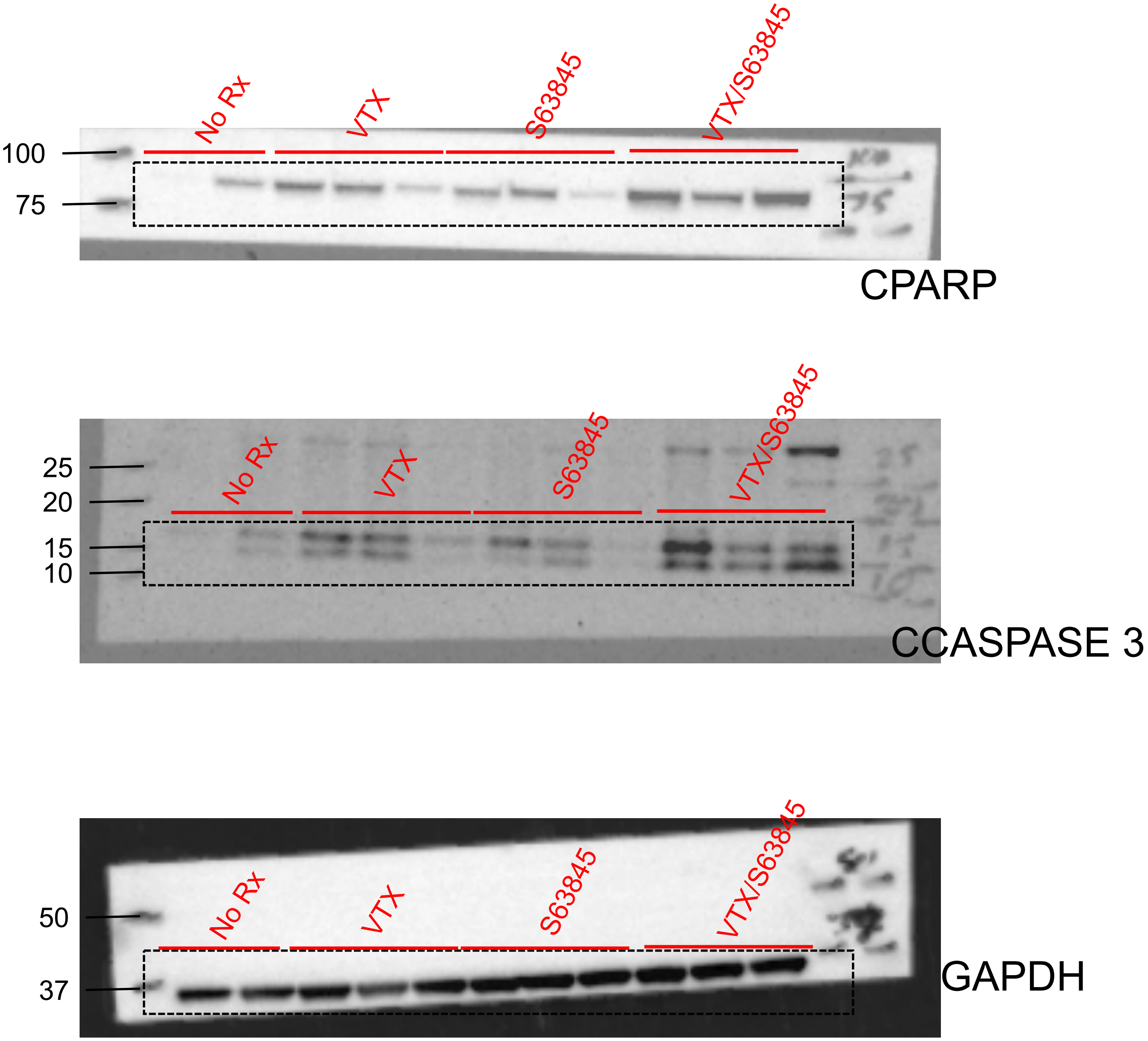
No Rx
VTX
S63845
VTX/S63845
100
75
CPARP
25
20
15
10
CCASPASE 3
50
37
GAPDH

Supplemental Figure 1B

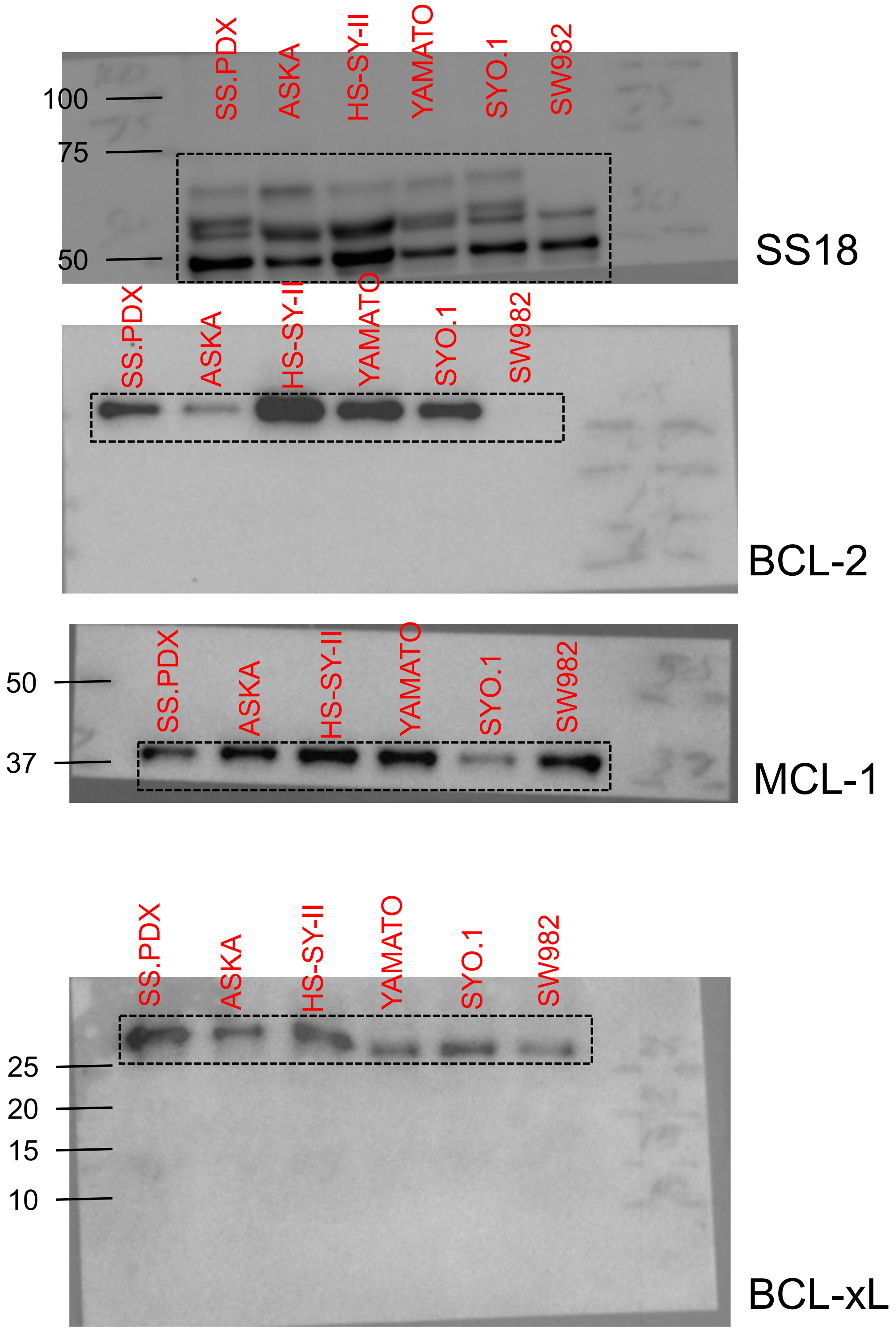

Supplemental Figure 1B

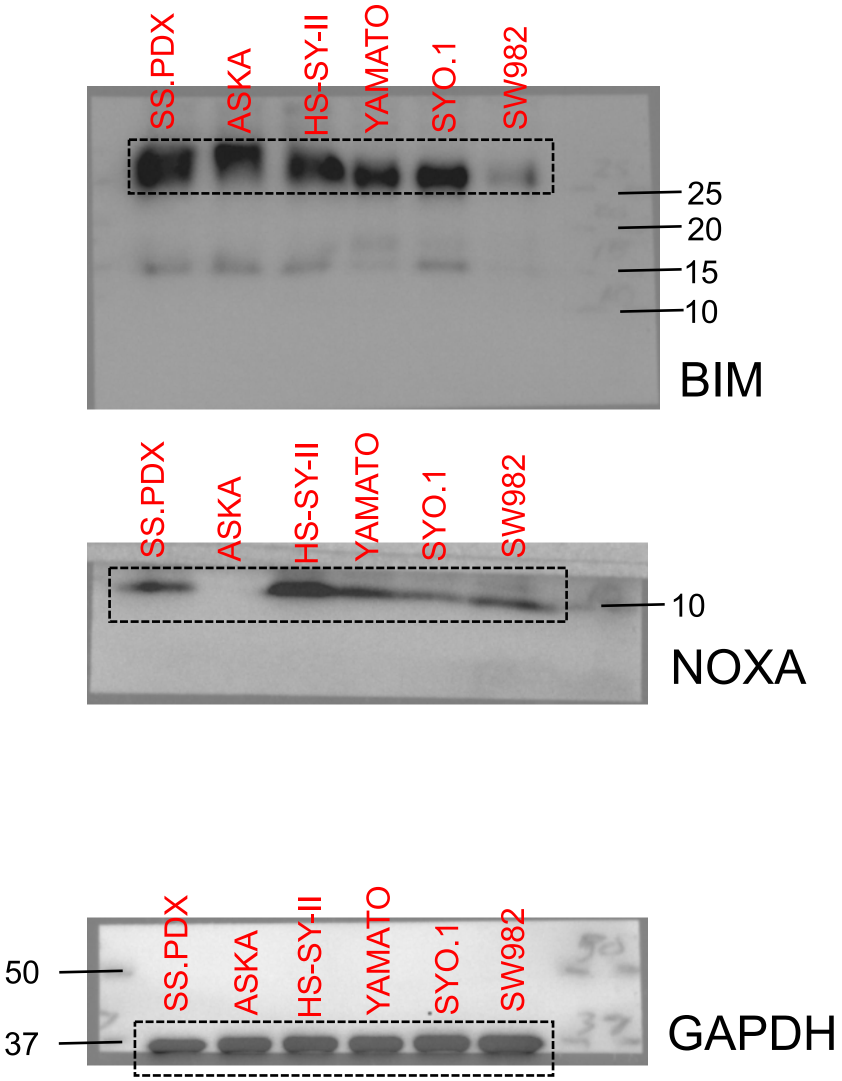

Supplement: Supplementary file 1 [file cancers-13-02310-s001.zip › cancers-1150036_supplementary material.pdf]
