# Supplementary figures and images for: Unmasking BCL-2 Addiction in Synovial Sarcoma by Overcoming Low NOXA
Source: Cancers (Basel). 2021 May 12;13(10):2310. doi: 10.3390/cancers13102310 (PMC8150384; doi:10.3390/cancers13102310)

**A**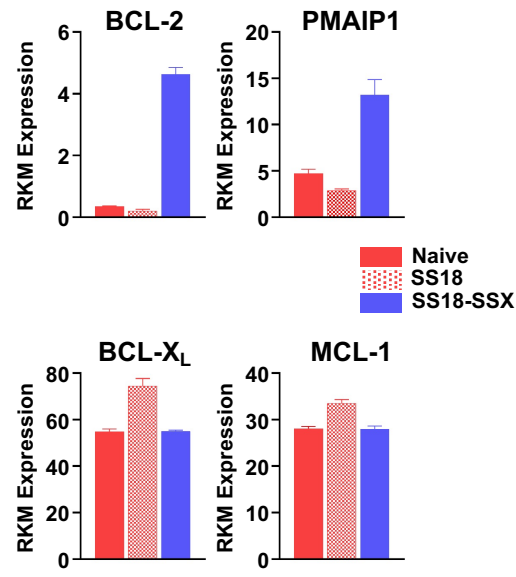**B**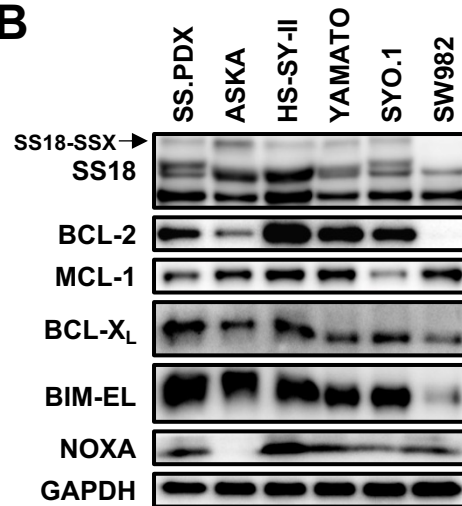

Supplement: Supplementary file 1 [file cancers-13-02310-s001.zip › Figure S1.pdf]

**A**

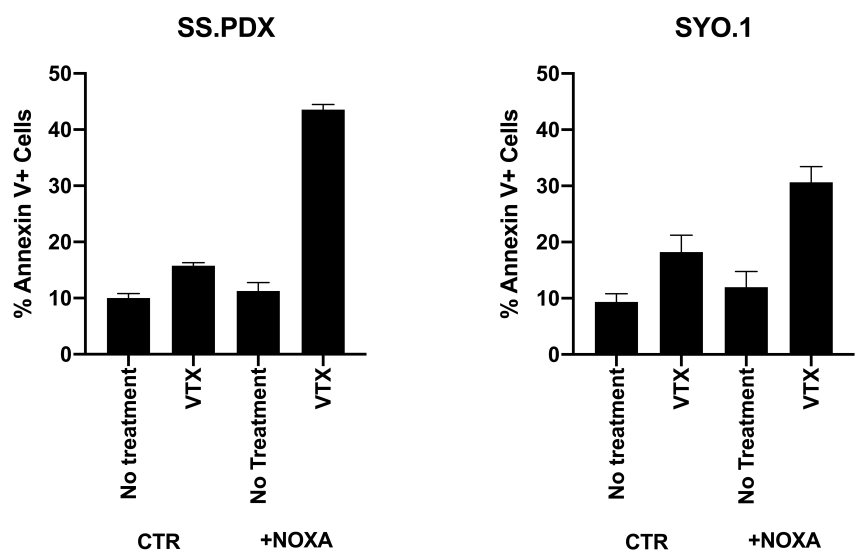

**B**

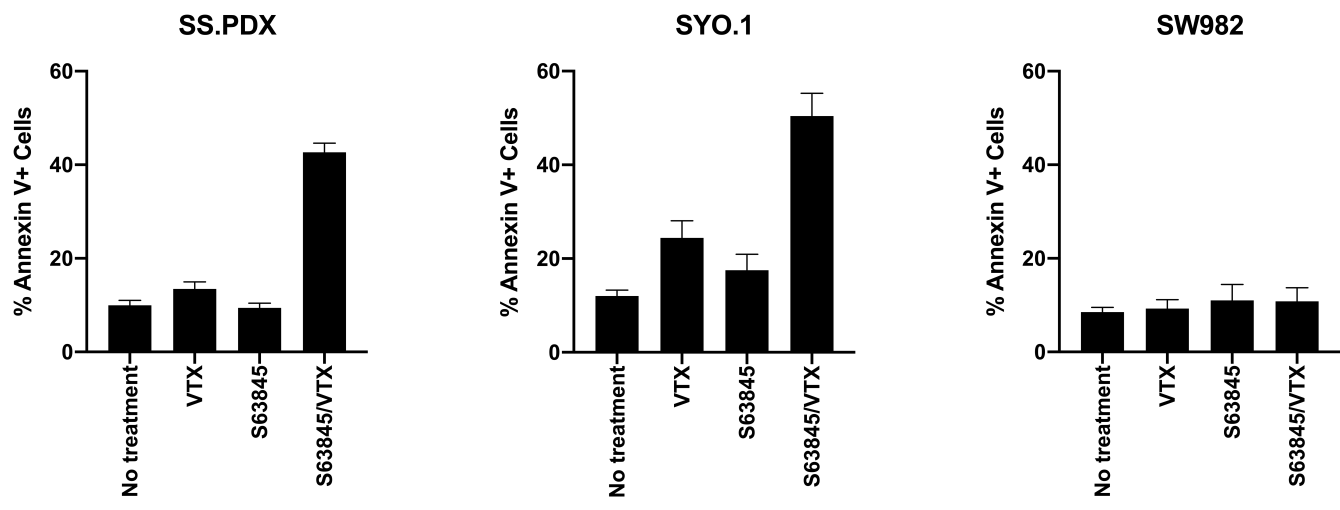

Supplement: Supplementary file 1 [file cancers-13-02310-s001.zip › Figure S2.pdf]
